# Foreign

**Published:** 1840-12-12

**Authors:** 


### FOREIGN.

### M. GENDRIN ON UTERINE HÆMORRHAGE.

(Concluded.)

M. Gendrin notes the disappearance of varices from the limbs as inducing uterine discharge; and also remarks the tendency of hot, hip, or foot baths, drastic purges, bleeding in the foot, &c., as producing flooding.

Our author next passes to the consideration of diseases of the uterus as causes of hæmorrhage. He relates that it occurred once after inflammation, once where fibrous tumours were contained in the uterine cavity, and in a third case where the cervix uteri was much enlarged; but the most frequent of this class of causes, he thinks, is inflammation of the ovaria and Fallopian tubes; indeed he would connect this state of these organs and uterine flooding almost as a constant conjunction. We ourselves have found that "the elongated cervix" is both accompanied by menorrhagia in the unimpregnated and flooding in those who have become so in a few instances.

The influence of polypus has been very marked in the two examples where it existed as a complication of labour—though in a third case, in which the polypus was of great size, no flooding took place. We have known several examples in the practice of others of very large fibrous tumours, not causing inordinate discharge. As to inflammation of the ovaria, Boivin attributes one class of abortions to the adhesions formed by the Fallopian tubes hindering uterine expansion. We have not remarked the coincidence between flooding and inflammation of these organs, or perhaps we have overlooked it. There are many physiological facts, however, which would incline us to receive it, if not with all the exclusive faith of our author, yet with much.

The anormal insertion of the placenta is, according to our author, a very frequent cause of uterine hæmorrhage. Madame La Chapelle says it is the sole cause of flooding in the latter months of gestation. Rigby, of Norwich, found, n one hundred and six of this last class, forty-six resulting from the anormal implantation of the placenta near the os. According to M. Gendrin, unless this position of the placenta be very obvious, it is usually overlooked by accoucheurs.

*Proximate Causes.*—The early hæmorrhages have for the proximate cause the disruption of the vascular connexion between the decidua and womb. Both deciduæ, the true and the reflected, are, says M. Gendrin, vascular. In spite of M. Velpeau, we too adopt this view, and have tried the experiment of W. Hunter of spreading a piece of these membranes on a sheet of paper and holding it to the fire, to show us, in its black streaks, that blood coagulated which had flowed in its very thin vessels. These very early hæmorrhages are not unfrequently thought to be a retarded menstruation bursting out with more than ordinary violence precisely because of the retardation. If they occur in married women previously quite regular, or if a woman has been regular both as to time and quantity of discharge, two or three consecutive months, and then there follows a retardation as to time, and an increase as to quantity, it is advisable to treat such persons as having suffered early miscarriage, in order to give them a chance of gestation as well as of conception.

M. Gendrin gives us three ingenious conjectures as to the causes of the anomalous implantation of the placenta: 1. The orifice of the Fallopian tubes may open nearer the os than the fundus uteri—a fact observed by him in the dead body; he nevertheless does not attach much weight to his own conjecture, neither do we. 2. The ovum may glide too low. 3. The thick mucus contained in the Fallopian tubes may project too much behind the decidua, and so permit the ovule to descend.

From the second to about the twelfth to the fourteenth week, we know that the deciduæ are approaching each other until they coalesce at the last named period. During this time the circulation in the maternal vessels of these membranes is ample: hence any of those causes which act in the production of menorrhagia cannot fail to influence the chances of uterine hæmorrhage at this point of gestation.

From the twelfth week a double vascular organization is going on, in the formation of a fœtal and a maternal placenta. On the maternal side the decidua serotina is thicker and more vascular than any other part of the caduca lining the uterus, and on the fœtal side the umbilical vessels have begun to climb into the villi of the chorion, to form many tortuosities, and twistings, and knobs. In this way the two systems of circulation mutually interproject, without ever commingling directly their several fluids. This early type of the placenta is the permanent formation in the horse, in which animal we have repeatedly seen that the chorion, vascular in all its surface, is in juxta-position merely with the vessels of the lining membrane of the uterus; that its vascularity is equally diffused, and that the union between them is just what may be represented by the apposition of two wet cloths.

At the middle period of gestation in the human female the maternal placenta is more than a third of an inch thick. Its vessels have two peculiarities: they are first very thin—so thin as to be, according to some, mere prolongations of the inner membrane of the uterine veins. According to Stanley and Mayo, the column of maternal blood is carried into canals formed by thin projections of the decidua. Whatever opinion be adopted, this is true—that the decidual vessels are very thin. The second peculiarity is that they expand into pouches at their extremities. The circle formed by the line at which the decidua vera becomes reflected into the decidu reflexa, constitutes the limits of the placental discs; here the union with the uterus is most intimate. This collar, conterminous with the edges of the placenta, is more solid in the earlier months; hence the known difficulty of abstracting the placenta in abortion. It becomes less intricate as gestation advances; hence the comparative facility of removing this organ. In the early periods of placental formation, the maternal disc is as thick as—M. Gendrin says thicker than—the fœtal, but gradually becomes thinner towards the latter end of gestation; from which circumstance he would deduce the cause why the fœtus is more independent of hæmorrhage in the end than at the beginning of pregnancy.

*Uterine turgescence* is next viewed as a proximate cause of flooding, or as M. Gendrin calls it in his margin, "cause immediate." Now, though there cannot be two immediate or proximate causes of any effect, still we may look on the expression as indicating a condition without which flooding could not exist—the "causa sine qua non" of logicians.

The first change determined by conception is afflux of blood into the whole generative apparatus from the ovary to the os tincæ; and this afflux continues with such unceasing vigour that in the last months of gestation the uterus may be looked on as an erectile tissue, between the internal and external strata of muscular fibre, of which a layer of thick, very short, but capacious veins is interposed. These veins are so short as to pass for cells; but they resemble exactly the distribution of the cells of the corpora cavernosa penis, which Tiedemann has proved to be a congeries of veins. The arterial distribution is also copious, and may, at a glance at William Hunter's plates, be thoroughly appreciated. The termination of these veins on the fœtal placenta and the decidua, their size, so expressively named "sinus," their number, friability, present to the student an apparatus for circulation, in any derangement of which he will readily apprehend why the effect should be so terrible as to have deserved, even at the hands of the common observer, the appropriate appellation of a "flooding."

We may fairly then grant that with such a supply of blood, and with such an arrangement, the vascularity of the uterus must be considered as an immediate cause of its hæmorrhage. M. Gendrin after giving a very incomplete description of the uterine muscles, seeks in their spasmodic action for another influential agent in hæmorrhage. Spasms are irregular contractions; irregular contractions may tear up the placental or decidual organs; hence hæmorrhage: such is his position, strengthened by the fact that the patient experiences not unfrequently violent movements which precede the visible or the latent flux. We admit in theory this cause. In practice we have known uterine spasms occur for months daily, in spite of large opiates constantly administered; and rarely, indeed never, have we seen them occasion flooding. The agency of the muscularity of the uterus is especially preservative: these fibres are the natural ligatures to vessels, which without their contraction would sluice away life in a very few seconds: still, however, we admit the theoretical probability of irregular uterine contraction being followed by hæmorrhage during gestation. After childbirth, irregular contraction, in its broadest acceptation, combined with partial or total separation of the placenta, is indeed the commonest condition of flooding. We differ with M. Gendrin when he states his disbelief of the uterine spasm, so often noticed by women as "violent motion" of the child, being really in most cases actual fœtal movement: we have, in the severer complications of labour, in convulsions, in hæmorrhage, felt this fœtal movement and known it to precede the death of the infant; while, in other examples of flooding, we have had every reason to believe that the flooding *preceded* the movement, which though it certainly existed as uterine, was caused by and did not cause flooding.

*Placenta prævia.* M. Gendrin now proceeds to consider how hæmorrhage takes place in placenta prævia, (p. 267,) and the speculations are to us new and curious. It is usually thought that in these cases the necessary expansion of the cervix uteri, in the latter months of gestation, tears away the connexion between the placenta and womb, and thus freeing the uterine sinuses, gives rise to "unavoidable" hæmorrhage. M. Gendrin, however, takes just the opposite view, and asserts that the flooding is owing to ruptures, not of the uterine vessels from the edges of the placenta, but of the central parts of this last organ only. According to him, the circle sketched out on the womb by the reflection of the decidua vera into decidua reflexa, is the limit of the edges of the placenta; that this organ never overslips this limit; that the expansion of the womb increases the capacity of this circle without altering the original fixture of the circumference; in a word, that the placenta does not grow by extending its edges, but by increasing its centre in the increased space formed by the expansion of the fundus uteri. Now, if the placenta be fixed, from first to last, to the cervix uteri, as long as the cervix dilates and is accompanied by just as much placental development as will fill the increased uterine space, no hæmorrhage will ensue; and this happens during the first half of gestation, when, though the cervix uteri begins very early to expand, still no bleeding occurs. In the latter half of gestation, however, the neck of the uterus does not expand so rapidly as the placenta grows: the result is "that the placentary adhesions are necessarily destroyed from the centre towards the circumference of the cervix. The mucous membrane of this last part is separated from the placenta by a mere layer of mucus; then it is, that there are transudations from the placental vessels. If the placenta be attached by its edges to the cervix uteri, the separation of the sides of the neck breaks up the placental cotyledons; as much by the distension they suffer, as by the endeavour to become engaged in the cavity of the cervix." (p. 269.)

If our paraphrase of M. Gendrin's views be correct, he believes that the bleeding in placenta cases is from ruptures in, or exudations from the placenta almost always, and not from denudations of the uterine sinuses. The anatomical details already given of the state of the placenta in such cases, if exact, would afford some colour to M. Gendrin's speculations. Still we are inclined to retain our former belief, that the source of flooding in placenta prævia, is from this organ being torn by uterine expansion or contraction from the womb, and not from a rupture of the central parts of the placenta itself. It is true that many of the phenomena of these miserable cases will be explained by either theory; that, for example, the gushes attendant on labour-pain, and ceasing when the uterus ceases to contract, may equally exist under a supposition that the placental rent is further lacerated, or the coagula stopping it removed by the expansive action of the os during uterine contraction; as by the old supposition, that this contraction has created fresh separation between the vessels which formed the connexion between the womb and the placenta. Under either theory, we say, the remittent gushes coincident with remittent uterine action are accounted for: but surely the mass of blood lost is maternal, which would scarcely be the case if it came from a rupture of the after-birth: the very tangled and interlaced texture of this organ makes the flux from rupture comparatively slow. We must remember, too, that the placenta is made up in bulk by fœtal vessels, among which the deciduous maternal vessels are placed; hence the escape of any one infant, in ruptured placenta, is, under the theory of M. Gendrin, scarcely possible, and yet a certain number are always saved in placenta presentations. We have delivered both by perforating the placenta in its centre, and by separating its edges, and have found the flux of blood much greater in the latter than in the former cases; so much so, that where the mother had already lost much, we have invariably on the grounds of a less, and a less rapid hæmorrhage, preferred perforating the placenta, to separating it, in cases where the implantation has been centre to centre.

*Diagnosis of utero-placental hæmorrhage.*— In all doubtful but important cases, it is necessary to make a thorough examination, and not to hesitate to introduce a part or the whole of the hand if requisite. Utero-placental hæmorrhage may be distinguished from menstruation during pregnancy, by the presence of pain in the former, and its absence in the latter; by the periodicity and by the quantity of discharge. Sometimes there is a varicose state of the vagina, which would be liable to mislead those who do not examine the state of the organs themselves.

The diagnosis of placenta prævia, M. Gendrin says, is not so easy by the touch as books would make us believe, and we agree with him. In the first stages of labour, before the os uteri is much open, the kind of hæmorrhage has been our guide to suspicion. Clots entangled at the os uteri prevent a due recognition of what is behind them; and mere curiosity must not have place in a case where the removal of a clot may be followed by a fatal gush; still, the absence of the resistant sensation which the fœtal head affords, even through the uterine walls, coupled with the kind of hæmorrhage, is generally deficient in the last weeks of pregnancy, to leave few doubts as to the case. When, during early labour the os uteri is open, the unequal spongy substance of the placenta is readily, we think, known; but M. Gendrin adds a very valuable sign of diagnosis, if it be well made out, viz., that there will be felt a pulsation at the os uteri, not synchronous with that of the mother's pulse, but with the quick measure of the fœtal heart. We have never as yet tried this: indeed, in placental cases, he who takes the earliest period in which it is possible to deliver, will be the safest practitioner, and we rarely could venture to do otherwise. A second mark is the impossibility of producing *ballottement*, which M. Gendrin found in three examples: he, however, does not assign this as positive. We know of another instance corroborative of this diagnostic symptom. An eminent practitioner, now dead, being consulted in a case of doubtful pregnancy, detected every sign but that known under the name of "ballottement:" though, therefore, he could be sure of an enlarged uterus, he could not say positively whether such enlargement was owing to the presence of a fœtus; he gave it as his opinion, that if the woman were pregnant, the placenta was interposed between the cervix uteri and the head of the child. This sagacious surmise eventually proved quite true. Before, however, the ballottement be taken as a test of placenta prævia, and before the practitioner should indulge himself or the friends of the patient, in the gloomy anticipations attendant on such examples, he should be aware that this bounding movement given to the fœtal body floating in liquid, can only be produced with certainty between the five and a half and seven and a half months of gestation. Even then there must also be that adjustment between the developed weight of the fœtus and the quantity of liquor amnii, that the former shall be so nearly of the same specific gravity as the latter, as to be readily made to ascend and descend. If this proportion be not exact, and the fœtus be too heavy, it cannot be moved: and if too light, it will not descend; in either case the ballottement is imperfect or null. The stethoscope gives, we think, in placenta cases, a very doubtful diagnosis. In Dr. West's very neat translation of the younger Naegele's work on Obstetric Auscultation, it is asserted that in placenta prævia the murmur is heard loudest over the pubis. This sign can but be considered as an adjunct to others; it is very deceptive by itself.

The history of the case is, however, the most valuable diagnostic element. "Flooding, without pain, at decreasing intervals prior to labour, and flooding coincident with uterine pain, in labour," will be sufficient grounds for the strongest suspicion.

The diagnosis of internal hæmorrhage is most difficult and most important. We have already slightly noticed the extreme value of changes of respiration, sighing or yawning, as indicating, prior to syncope, the presence of concealed flooding. Latent hæmorrhage, in the early months of gestation, may be inferred from the following group of symptoms: sudden uterine spasm, a shivering fit, cold extremities followed by syncope actual or impending, accompanied or succeeded by "*tenderness* on pressing the womb." This condition ends by a discharge by the vagina of serum slightly tinged, and by slow recovery from fretful fever; or by a continuance and even aggravation of this fever, with a cessation of abdominal expansion, so as to lead to suspicion of the death of the fœtus.

In the latter periods of gestation when the womb is distensible, the quantity of blood effused being greater produces more marked effects—sudden syncope, or sudden diminution in the force, with increased rapidity of the pulse: then follow the effects of the loss of blood—the pallid cheek, hollow eyes, limbs bedewed with cold sweats, hurried respiration, great terror, and great helplessness. If now the hand be laid on the abdomen, the womb will be felt soft, doughy, obscurely fluctuating, flaccid, and *suddenly enlarged.*

Intermittent internal hæmorrhage may be suspected by the presence of spasm of the uterus, by more or less of syncope, and the continuance of feeble health, only broken in on by those storms which threaten to sweep away the double life.

Latent hæmorrhage, *during* actual labour, is known by the above symptoms of syncope and coldness, followed by the sudden diminution of the pains; by increased uterine bulk during the cessation of uterine contraction, and especially by that diagnostic of Levret, pain on pressing the uterine tumour.

*Treatment.*—Our author divides cases of uterine hæmorrhage into two classes: in the one those accidents have not as yet occurred which put a period to gestation; in the other, it is impossible to prevent the expulsion of the uterine contents. In the former cases the object of medication is to prevent labour; in the second to hasten it; while, at the same time, every means is resorted to stop the flooding. In the first class, then, of cases, the first thing to do is to allay every symptom precursive of excessive uterine plethora. We are not to wait till there has actually been a flux before we act; it is then often too late; but our prophylactic treatment should commence with well-grounded suspicions of disordered circulation in the womb. The rules laid down by our author on this head are abundantly strict:

"Abstract even the slightest causes tending to keep up uterine congestion; remove every band which restrains the current of blood; let the patient assume the horizontal posture, from which she is to swerve neither for the wants of nature nor the toilette; the pelvis must be raised above the level of the rest of the body; the temperature of the room should be rather cool than hot; all causes which act directly on the uterus as well as all moral influences should be abstracted; all objects of undue interest, and all intellectual occupation must be eschewed; the diet should sustain but not excite, so that the digestive organs should be called into a feeble activity only; the alvine secretions should, however, be sedulously regulated." (p. 306.) Such are M. Gendrin's directions: an excellent mark to aim at certainly; but he who expects to effect such a change in the excitable habits of the upper classes, as to induce them thus to vegetate, ought to furnish us with a recipe to control the ennui which preys on minds which are told to banish all feeling and all intellect. These little exaggerations of M. Gendrin are pardonable, however, and may be even commendable, if rightly appreciated, as including good directions for keeping the body and mind quiet, without attempting to torment the latter while you imprison the former. These means, says M. Gendrin, should be enforced for twelve weeks, when the placenta has a more developed energy and hold. If, however, the flooding continue in the fourth month, the same regimen must be enforced until the last month of gestation.

Bleeding is discussed cautiously by our author. He allows that it is a provocative of abortion injudiciously used, while, on the other hand, it saves the plethoric from this result if timely employed; even in those uterine irritations, the common result of the congestion of pregnancy, he does not scruple to recommend the authority of Mauriceau who, in one case, bled eighty times during gestation. We are certainly one of those whose experience is not favourable to the use of this remedy in gestation. The cases must be well marked indeed which require general bleeding; on the other hand the greatest amount of plethoric ailment in the mass of women with child, yields to a cupping on the loins or to leeching. The nervous shock in the excited state of system induced by pregnancy is very great among the wealthier classes, and cannot be disregarded but at a woful sacrifice. Among the hard-fibred and indifferent, in cases, too, where there is no doubt as to uterine as well as general plethora, venesection may be demanded, but it should never be resorted to unless the indications be most positive. M. Gendrin, however, by stating the dangers of over-bleeding or of injurious bleeding, has neutralized the "verba magistralia" as to its employment. He puts forth prominently the increase of nervousness, the constant headach, the perpetual febricula, the deranged digestion, the uterine spasms reproducing uterine hæmorrhage, the subsequent difficulty, not to say impossibility, of combatting these "symptoms caused by the lancet" with any hope of success. (p. 312.) To these observations there are appended others indicating the great danger of inducing syncope in pregnant women; the necessity, therefore, of never bleeding but by a small aperture, and in the horizontal position; and, further, of never reiterating the emission save it be ascertained that the process of blood-making is rapid and certain.

With regard to the indications for local bleeding, our author names the constant presence of lumbar pains and the appearance of varices and hæmorrhoids. Care must be taken to shun producing syncope, to avoid, also, bleeding when there is no local turgescence, as then the local emission produces, by attraction, local congestion.

*Irritants* are liable to less objections than depletants, according to M. Gendrin; hence he quotes Riverius as recommending sinapisms, dry cupping to the thorax, mammæ, hypochondria and abdomen. Care should be taken, however, not to over-excite the nervous system; hence irritants rather than vesicants are to be employed.

*Sedatives* are a dominant indication in uterine flux. Having given repose to body and mind, cold should be used; and, first, the drinks should be all cool before they be gradually reduced to the temperature of ice. The solid food should also, according to our author, be cold. Cold, sponging, tepid cataplasms about the abdomen and loins, with the derivative treatment, are among the most successful means of arresting hæmorrhage. The cold hip-bath, too, is very useful; but the temperature should be cautiously regulated to the feelings of the patient.

Our author having asserted that ovaritis induces uterine flux, recommends general or topical bleeding when, during pregnancy, these states are coincident; remarking, however, that the disorder of the ovary being kept up by the natural plethora of pregnancy, is rarely removed till the latter months.

If these means do not succeed, M. Gendrin makes an issue ("exutoire profond") at the lower part of the linea alba—a practice which we would by no means imitate or recommend. The distinguishing the symptoms of a turgid ovary from those incident to a plethoric uterus must be a nice point; the constant coincidence of uterine flux and ovaritis is yet to be proved; the only part certain, then, is the torment of a profound issue over the pubic region, inflicted by a gentleman who, three pages before, is recounting the dangers of simple irritants on the susceptible nervous system of the pregnant female.

There remains one other set of remedies which, added to the abstraction of blood, the use of cold and of revulsives, complete the treatment of the first class of cases, viz., *antispasmodics*: of these, opiates are the chief. "We are not acquainted," says M. Gendrin, "with any therapeutic agent meriting more confidence than opium, whenever the uterus during gestation becomes the seat of spasmodic pains; nay, we never wait for the appearance of those shocks which give rise to these in pregnancy, and we resort to it whenever even there is an irritable nervous system." (p. 328.) Even when this valuable drug does not arrest it will mitigate the discharge. Smellie resorted to opium in all the serious hæmorrhages of gestation, early or late. Twenty or thirty drops of laudanum will immediately arrest, says M. Gendrin, a flow of blood which threatens to throw the patient into alarming debility, portending an immediate fatal termination. In this praise of opium we most cordially join. There is scarcely a case of inordinate flux in which it is not the best remedy, save in actual labour, when it is only second to manual aid, which, let it be emphatically understood, is in these cases the first. In pregnancy the usual dose of opium may be advantageously increased, and its effects should always be sustained by a regular repetition of it. A more direct effect is produced on the uterus if it be administered as a lavement, while the stomach and liver are not disordered as by the usual mode of taking this medicine. M. Gendrin combines opium with syrup of orange peel, extract of quina, sulphate of quina, gaseous alkaline drinks, for the purpose of remedying these effects on the stomach. This practice of Hoffman and Riviere is of singular benefit, and enables the practitioner to continue for months the use of opium with great advantage.

With regard to the second class of cases, those in which gestation is interrupted, the chief indications are as to the means by which the flux is most speedily quelled, all considerations as to the preservation of the gestatory state being banished from our mind. If the child have been killed by the hæmorrhage, but labour has not come on, the flux usually ceases; and though we do not state it so peremptorily as M. Gendrin, certainly there is not much hazard of a recurrence of flooding after the death of the fœtus, The labour at a later period is generally unhæmorrhagic; hence in these cases the indications are to guide the constitution through that group of morbific influences which we have already described as attending the presence of a dead fœtus in utero. Premature labour should rarely or never be induced in these examples. M. Gendrin says that the woman, whatever may have been the actual loss of blood, need not, after the blight of the fœtus, be kept supine, except, (he adds) in those very rare examples of a continuance of the flux. Where the intra-uterine flux has been extreme, inducing extreme debility, the indication is, according to our author, to gain time until the forces of the woman shall have been recruited to withstand the chances of a renewed hæmorrhage during labour.

If the hæmorrhage take place in the early periods of gestation, the uterus not being capable of containing a quantity detrimental to life, the woman is not in danger so long as the flux is not external; hence, in the early months of pregnancy, those means must be resorted to which restrain flooding within that limit, so that it shall not become external; and we must wait the expulsion of the detached ovum by nature, and never, within these limits of internal hæmorrhage in a small womb, must we provoke labour.

If after the fifth month of gestation flooding occurs, the effect is much more decided on the mother. If it be intense, the patient may succumb in a few minutes. These are cases never to be left until the hæmorrhage ceases, or until the fœtus is expelled or extracted. When the flooding is external there is not so much chance, says M. Gendrin, of utero-placental lesion as when it is internal: there are examples, and these not rare, where after such a gush the woman has still brought forth at the full period a live child. But he wisely adds that these instances should not lead to the adoption of the expectant method, by the hope of similar results to ourselves. The dictum of old Mauriceau should form the rule, a deviation from it the exception: "At what period soever of gestation the flooding exists, whether early or late, the most expedient and most salutary remedy for the safety of the mother and the infant is to deliver as quickly as possible. When the blood is so abundant in its flow as to throw the woman into frequent faintings and convulsions, the operation is not to be deferred; it is absolutely necessary to deliver her whether she be at her full time or not, or whether there be pains or not; unless, by neglecting these sole means of safety, you will be content to witness the last wave of blood gush out at the same time with the last sigh."

There remains for the most dangerous case the operation of *forcing* the uterus and turning the child. Forcing is a term, however, rarely realized in English obstetricy. In hæmorrhage, the os soon becomes flaccid, and requires no force for its dilatation. In those cases in which the delivery need not be instantaneous, M. Gendrin recommends, in common with most authorities, the rupturing the membranes, a practice first, we believe, adopted by Puzos, who waited for the bulging bag of the amnion before he punctured; while Smellie recommends the operation to be done as soon as possible. M. Gendrin says little or nothing on this very valuable remedial measure. Its merits, however, have been denied by some and exaggerated by others. Let us be distinctly understood, however, that the great—nay very great—majority of cases of flooding *during* labours are successfully treated by this mode of proceeding. Smellie, Baudelocque, Rigby of Norwich, and Merriman, have all given their assent to this practice. We believe that Hamilton of Edinburgh rarely recommended it. Of this, however, we only speak as former listeners to his lectures, the best we ever heard on midwifery. The exaggerators of the efficacy of puncturing the membranes as a remedy always successful have to answer for its indiscriminate use. It is not safe where the flooding is violent; it is not safe where the patient is much enfeebled; it is not safe where the fœtus does not present in its long axis; and Boivin would add, that it is not safe where there is little liquor amnii and a large child, for the uterus then cannot contract—the sole object for which the membranes are ruptured. In those cases of hæmorrhage during labour anterior to the rupture of the membranes, the examination should be made with the whole hand if requisite, and every point thoroughly determined before we proceed to any course of action. It is not always easy to perforate the membranes which in the commencement of labour often fit the child's head like a scalp. There has been a question whether plugging the vagina in cases of the kind now under discussion is not safer than rupturing the membrane. The quantity of blood pent up within the uterus, say the advocates of this mode of proceeding, must necessarily be slight, while the presence of the clots in utero, and of the "tampon" in the vagina, invariably produces labour-pains. So that while we preserve the membranes entire, we remove all those risks which arise in labours from premature evacuations of the liquor amnii. We confess we never have had the courage to attempt this course of action in similar cases. The reasoning is plausible, nay more, it has been supported by the practice of Baudelocque and others. In cases of placenta prævia, where we cannot dilate the orifice of the uterus, plugging is the only remedy; and it is so efficacious, that Wigand advises us to resort to no other, giving six or eight instances of the child having been born quite by natural efforts, as guerdon of his success. If plugging should be determined on, the operation should be so exact that not a drop of blood should escape externally. We never quit the bed-side whenever we have been induced to resort to the "tampon," and we would advise the practitioner to attend both to the rational signs of hæmorrhage as well as to the sensible one of a *distending* abdomen, and to act accordingly.

We need not, we presume, guard against our meaning being misunderstood, by stating that plugging is only to be resorted to when the uterus will not contain blood enough to injure the mother, consequently it never should be used *after the birth of the child* from the seventh month to the full period. Before the birth of the child, however, and before the rupture of the membranes, the case may occur in which the "tampon" is our sole efficient remedy; and then it is highly probable, though we believe not certain, that the womb will not admit of much further distension without immediate re-action. We say not certain—because we have known the blood burst through the membranes into the amnion, and we have cited examples of its being accumulated in the centre of the placenta. These, however, are rare cases, and ought to make us act with caution while we retain the operation of plugging, not as superseding that of puncturing the membranes, but as suited to peculiar states.

As to the mode of action remedial of the hæmorrhage incidental to abnormal implantation of the placenta, the following important observations of M. Gendrin must be stated in his own words:

"Hæmorrhage always ceases as soon as expulsive labour-pains commence, in cases where a portion of the placenta only is fixed over the os uteri. (Smellie, t. ii. p. 247 and 356.) The same result takes place where the centre of the placenta is over the os, as soon as the expulsive uterine efforts have ruptured the amnion. Flooding only recurs if there be inertia uteri. We are astonished that this remark has escaped the greater number of accouchers. If a gush of blood succeeds each uterine contraction, it is only in the first period of labour, and until the uterine contractions have become thoroughly established, in cases where the placenta is attached only by a segment to the os. When the attachment is centre to centre, then the flux continues up to the bursting of the amnion. This has been the course of matters in every case which we have seen. Hence we always hail the advent of true labour in placenta cases. We would thence also further deduce this point of practice, viz. the necessity of producing labour artificially whenever the flooding is in quantity or by frequent recurrence likely to injure mother and child." (p. 348.)

This recommendation is precisely what is affected by all accoucheurs, when in these terrible examples of flooding they turn the child; but this, the usual course, is not the one which M. Gendrin advises. He resorts to a method which he has tested by actual experience as capable of saving both mother and child. The plan is, to evacuate the liquor amnii by passing the requisite instrument through the placenta and so puncturing the membranes. M. Gendrin has resorted twice to this operation, and has been twice successful: Let us abstract these instances.

"Case I. A fair young woman had a gush of blood at the sixth month, and three more during the seventh; for each of which she was bled by her attendant, and thus reduced by the double loss to a state of debility so extreme as to make the upright posture in bed a cause of fainting. M. Gendrin being consulted, declared the case to be one of anormal implantation of the placenta, and recommended the puncturing the membranes, declaring that another gush of blood would kill her. The original attendant deeming the case one of mere congestion of the os uteri, resolved to plug the vagina, but while he was preparing the 'tampon' a fresh gush took place, which at once induced M. Gendrin to introduce his hand into the cavity of the pelvis. He found the cervix uteri rigid, and permitting him only to pass the tip of his finger, high enough however to allow him to recognize the placenta. His original proposition occurred to him of evacuating the waters by puncturing through the placenta. A female catheter—'une algalie ordinaire de femme'—was the instrument used, which, with the greatest facility penetrated the amnion through the intervening parts, and the liquor amnii immediately escaped. The hæmorrhage *ceased instantly;* but labour-pains did not take place till three hours after the operation; and in four more a child was born alive, which protruded the placenta before a part of its head and face. The uterus contracted well; a slight hæmorrhage only occurred, which in the debile state of the woman threw her into a fainting fit which lasted fifteen or twenty minutes. It was two months before the patient could leave her bed. Ultimately both mother and child did well. The placenta when examined was found perforated about two inches from its margin: blood was incorporated with its tissue in a space extending over two or three inches of its surface, in the centre of which the perforation existed.

"Case II. In this case, the puncture was made in the seventh month of gestation. The 'algalie' penetrated the placenta obliquely, entering about two inches from its margin and coming out about five lines from the edge of the fœtal surface. The infant died immediately after birth, but the mother not having suffered much previous to labour, experienced none of those accidents which occurred in the instances formerly related."

On these two examples M. Gendrin would base his recommendation to puncture, which, if not delayed too long, will, he says, be successful. Where it is not, and the uterus remains inert (the inertia he attributes to delay,) the practitioner may still turn the child. This method, he adds, has the advantage over the *accouchment forcé*, that it is applicable in any case, while the latter is, as we know, not always practicable before life ebbs away. We leave the further oonsideration of this method to our readers. That it is a valuable hint communicated in good faith and on high authority there can be little doubt.

That every aid to the practitioner in these terrible instances is a blessing, we acknowledge most thankfully. There is enough, both in the nature of the case itself, and in the source of the remedy, to make us carefully note the reasonings and facts, and to prepare us to attempt the same in similar circumstances. It is, however, untried, at least comparatively so, when opposed to the usual practice of immediate delivery by the hand. In the majority of cases, the mother is easily and instantly saved by this operation, and not unfrequently the child. Where this is practicable it should be done. The delay attending all other modes of proceeding, the dreadful anxiety of waiting for effects which we cannot calculate, the watching those points of time laden with the fiat of life or death, are persuasions to immediate action. There are occasions, however, in which the flooding is great, and yet the introduction of the hand impossible; and it is in these in which we think M. Gendrin's plan should be tried. We call it M. Gendrin's plan, because it is fairly and substantively put out as a determinate course of action in certain cases; still, neither the suggestion nor the practice is his. Mr. Ingleby of Birmingham has given us a summary of this practice, at p. 157 of his book, from which it appears that Dr. F. Ramsbotham recommends rupturing the membranes in partial implantation of the placenta over the os, that Dr. Cusack performed this operation in similar cases six times; and Dr. Blundell, also, has resorted to and advises it. M. Gendrin is distinguished from these by a more distinct and a more extended recommendation of the operation, whatever may be the position of the placenta as to the os uteri.

We have conned this remarkable volume, leaf by leaf, from beginning to end. That it is the work of no common man is apparent in each sentence. It abounds in a patient display of materials, and in views in which empiricism is contending with philosophy. There is much of reading, much of observation; a due deference to the opinion of others and an honest confidence in his own. The subject is encumbered by method, stifling the free play of thoughts by the logical armour which was intended as a help and a defence. In spite of these faults, however, the work is highly creditable to M. Gendrin and to the modern French school, which is striving to unite the wide survey of the patient German with the restless and searching empiricism of the English.—*Brit. and For. Med. Rev.*

---

*British Association for the Advancement of Science.*

*Glasgow, Sept.* 17.—Dr. Perry read a valuable paper "on the diffusion of contagious fevers, and on the laws that govern them." The paper was accompanied by statistical tables, compiled from several thousand cases, from which many valuable deductions were drawn. Dr. Perry denied that the enormous proportion of fever cases, occurring in Glasgow, depended on any circumstances of situation, air, or filthiness, in the town itself, but was owing to the diffusion of the contagion by the hordes of Irish and Highland emigrants. A large portion of the paper was devoted to the analogy between typhus and the acknowledged exanthematous diseases, viz., variola, rubeola, and scarlatina. This analogy he insisted on in the following particulars:—1st, it is generated by a morbid animal poison, which, once introduced, cannot be checked by any treatment; 2d, that it runs a definite course, terminating by a certain fixed period; 3d, that an individual once suffering from it, enjoys an immunity afterwards; 4th, that no other morbid poison can co-exist with it; and, 5th, that it is accompanied by a peculiar characteristic eruption. This eruption, he said, was frequently confounded with petechiæ, flea-bites, sudamina, &c., which occur accidentally in fever.

In its first appearance it is of a dull-reddish or rose colour, in the form of irregular spots; often slightly elevated, and felt by passing the hand lightly over them, but not acuminated. They can be easily made to disappear by pressure. They are unaccompanied by itching, are sometimes distinct, and occasionally so crowded as to give the skin a mottled or measelly appearance. It never becomes vesicular, and is sometimes so partial in its appearance, as to be readily overlooked by a careless observer. After a few days its colour fades, and it becomes of a dusky hue, like freckles, for which it might be mistaken. In bad cases of typhus it may assume a livid hue, and is then with difficulty distinguished from purpura.

The contagious nature of the disease was amply proved from the numerous cases; and the management of the sick and convalescent, so as to prevent its diffusion, laid down. The paper concluded with some excellent hygienic regulations, which, under an efficient system of medical police, would be highly conducive to the prevention of this species of fever.

The section adjourned at 2 o'clock.

*Sept.* 18.—Dr. Makay read a paper on the preparations of the Matias bark, the subject which he brought before the section last year. Dr. Makay exhibited drawings of, and read an anatomical description of a monocephalic double monster; it was the offspring of a negress residing near Maracaibo, in South America; it appeared to be born about the seventh month of fœtal life, and was said to have lived several hours.

Dr. Patrick Newbiggin, of Edinburgh, read a communication on the therapeutic effects of castor-oil in certain nervous disorders, in which he was desirous of showing that, independent of its well-known purgative property, the Croton oil possessed specific influence in epilepsy; and in the various forms of neuralgia, as in tic-doloureux, sciatica, &c. He was induced to form this opinion, in consequence of his experience of the treatment of such complaint, in his practice at the New Town Dispensary of Edinburgh, as well as in private practice. The author detailed some of the cases, more particularly of epilepsy, in which he had produced entire relief from that very grievous complaint, and mentioned especially one instance of a cure having been effected after the disease had existed upwards of twelve years. Dr. N. stated that he had remarked very decided benefit from this remedy in laryngismus stridulus, or crowing disease.

Sir Charles Bell expressed an opinion in the conversation which ensued, that this medicine is efficacious in those neuralgic affections when seated in the face, but will fail in those seated in the extremities, unless accompanied by some symptom manifesting cerebral disease.

Dr. Abercrombie bore testimony to its utility in affections of the brain, from long experience of it, and mentioned a remedy which he found very serviceable in laryngismus stridulus, viz. a combination of carbonate of iron and rhubarb with musk.

Dr. Lawrie read a most elaborate paper "on the results of amputation." In which the results of all the cases which had occurred in the Glasgow Infirmary for many years were stated, and all the great questions connected with amputation were ably discussed in connection with those results which were drawn from tables in which the operations were classified according to the sex of the patients; the cause of operation, whether disease or accident: the limb operated on, whether primary or secondary, &c. &c. The following are some of the results obtained. Out of 276 cases, 176 recovered, 100 died; 216 were males, 60 females, 153 were for previously existing disease, 123 for injuries. One case of operation at the hip-joint occurred, which recovered. Generally speaking, secondary amputations were preferred, and immediate dressings approved of. The causes of death in the fatal cases were classified, and were frequently found to depend on visceral disease.

From the importance of the subject and the value of the communication, the section recommended its immediate publication *in extenso.*

Dr. John Reid read a paper "on the anatomical relations between the mother and fœtus (human)." The subject was illustrated by diagrams on a large scale, and *demonstrated* by preparations in a natural and an injected state. The tufts of placental vessels were shown to penetrate the decidua, and enter the open mouths of the uterine sinuses. These tufts were shown to consist of an artery and vein terminating in a blunt point, and continuous. A number of these, not bound together by cellular tissue, forming each tuft, which was continually immersed in the maternal blood, poured into the sinuses by the tortuous uterine arteries, whose lining membrane expanded into the sinuses, and was continuous with the uterine veins; after being reflected on the tufts of the placental vessels, forming, in fact, sheaths for them, these tufts resembling the branchial vessels of aquatic animals, covered by a prolongation of the inner coat of the vascular system of the mother.

Dr. Reid communicated a paper on the physiological action of Bromine and its compounds by Dr. Glover.

*September* 19.

Dr. R. D. Thompson read a paper "on opacity of the cornea, produced by sulphuric acid." The principal object of which was to prove that the opacity produced by the application of the acid consisted of *a false membrane*, external to the transparent layers of which the cornea in a state of health consists, which may retain their transparency after the external injury has been inflicted; and that this false membrane may be removed by operation, leaving the exterior coat (the conjunctival) free from any permanent effect capable of destroying vision. The paper was illustrated by preparations and experiments on the eyes of lower animals, demonstrating the membrane and the transparency of the layers immediately beneath it, when removed.

Mr. Douglas read a paper "on dislocations of the ankle-joint, backward and forward." Cases which came under the author's notice were detailed; casts and preparations of the parts illustrated the descriptions.

The following are the more remarkable appearances in a case of dislocation of the tibia forwards. The ankle was extended, and quite stiff; tibia advanced three quarters of an inch; the anterior edge being exactly over the articulation of the astragalus with the os naviculare. The tendon of the tibialis anticus was in a straight line with its insertion. Behind, the astragalus projects so much that the flexor longus pollicis does not run in its groove in the tibia at all. The astragalus and os calcis are in their proper relation to each other, and their posterior ligament is entire. Additional ossification took place at the back of the tibia, close above the astragalus. The external malleolus remained perpendicular in its situation, with its three fibulo-tarsal ligaments entire. A hollow ran obliquely upwards and backwards, from its anterior edge, showing where a fracture had taken place, the superior anterior portion having been thrown forward with the tibia. Internally the deltoid ligament seemed to have been ruptured.

A case of fracture of both bones close to the malleoli, which simulated luxation backwards of the tibia, was detailed.—*London Lancet.*

